# Artesunate induces necrotic cell death in schwannoma cells

**DOI:** 10.1038/cddis.2014.434

**Published:** 2014-10-16

**Authors:** R W Button, F Lin, E Ercolano, J H Vincent, B Hu, C O Hanemann, S Luo

**Affiliations:** 1 Peninsula Schools of Medicine and Dentistry, Institute of Translational and Stratified Medicine, University of Plymouth, Research Way, Plymouth PL6 8BU, UK; 2 Soochow University School of Pharmaceutical Science, Suzhou 215123, China; 3 Plymouth University Peninsula Dental School, 16 Research Way, Plymouth, Devon PL6 8BU, UK

## Abstract

Established as a potent anti-malaria medicine, artemisinin-based drugs have been suggested to have anti-tumour activity in some cancers. Although the mechanism is poorly understood, it has been suggested that artemisinin induces apoptotic cell death. Here, we show that the artemisinin analogue artesunate (ART) effectively induces cell death in RT4 schwannoma cells and human primary schwannoma cells. Interestingly, our data indicate for first time that the cell death induced by ART is largely dependent on necroptosis. ART appears to inhibit autophagy, which may also contribute to the cell death. Our data in human schwannoma cells show that ART can be combined with the autophagy inhibitor chloroquine (CQ) to potentiate the cell death. Thus, this study suggests that artemisinin-based drugs may be used in certain tumours where cells are necroptosis competent, and the drugs may act in synergy with apoptosis inducers or autophagy inhibitors to enhance their anti-tumour activity.

Artemisinin, a sesquiterpene lactone isolated from the Chinese herb *Artemisia annua* L., has profound activity against malaria.^1^ Artemisinin contains an endoperoxide moiety that reacts with iron to produce toxic reactive oxygen species (ROS). When malaria parasite (*Plasmodia*) consumes iron-rich haemoglobin within its acidic food vacuole in erythrocytes, the exposure of artemisinin to haem-derived iron results in lethal ROS production that exerts fatal toxicity to the parasite.^2^ Therefore, artemisinin, its water-soluble derivative artesunate (ART) and other analogues are potent in killing malarial parasites.^1,3^

Cancer cells contain substantial free iron, resulting from their higher-rate iron uptake *via* transferrin receptors compared with normal cells. Therefore, artemisinin-based drugs such as ART possess selective toxicity to cancer cells.^4, 5, 6^ Importantly, the pharmacokinetics and tolerance of ART as an anti-malarial drug have been well documented, with clinical studies showing excellent safety. Collectively, these properties make artemisinin-based compounds attractive drug candidates for cancer chemotherapy. Artemisinin and ART have been shown to induce cell death in multiple cancer cells, including colon, breast, ovarian, prostate,^7^ pancreatic^8^ and leukaemia^9^ cancer cells. Preliminary *in vivo* experiments also indicate the therapeutic potential for these drugs as anti-cancer treatments. In animal models, artemisinin or ART has shown promising results in Kaposi Sarcoma,^10^ pancreatic cancer^11^ and hepatoma,^12^ while compassionate use of ART in uveal melanoma patients fortifies standard chemotherapy potential for the patients.^13^ Currently, ART is on clinical trial for breast cancer treatment (ClinicalTrials.gov ID: NCT00764036).

Programmed cell death (PCD) is one of the critical terminal paths for the cells of metazoans. Among PCD, apoptosis has been well studied and it is known that caspase activation is essential in this process.^14^ In addition to apoptosis, necroptosis is another form of PCD. The RIP1-RIP3 complex highlights the signals that regulate necroptosis.^15, 16, 17^ Artemisinin derivatives, mostly ART, have been suggested to lead to apoptosis *via* ROS production in cancer cells. Efforts have been focused on ROS-mediated mitochondrial apoptosis,^9,18,19^ and DNA damage^20^ in cancer cells. Recent data suggest that artemisinin and its derivatives may induce cell death or inhibit proliferation through diverse mechanisms in different cell types. Artemisinin or its analogues were shown to inhibit cell proliferation in multiple cancer cells by regulating cell-cycle arrest^21, 22, 23^ or inducing apoptosis.^24,25^ Nevertheless, the detailed molecular mechanisms underlying artemisinin or ART-induced cell death are poorly understood, thus need to be further addressed.

Neurofibromatosis 2 (*NF2*) is caused by the loss of *NF2* gene encoding Merlin protein. *NF2* gene mutations cause the low grade tumour syndrome, composed of schwannomas, meningiomas and ependymomas.^26^ All spontaneous schwannomas, the majority of meningiomas and a third of ependymomas are caused by *NF2* gene mutations. Notably, approximately 10% of intracranial tumours are schwannomas.^27^ Interestingly, *NF2* gene mutations are also found in a variety of cancers, including breast cancer and mesothelioma.^28, 29, 30^ The low grade tumours caused by *NF2* gene mutations do not respond well to current cancer drugs and therapy is restricted to surgery and radiosurgery.^26^ Therefore, there is a need for drug treatment of the diseases. Here, we show that ART sufficiently induced schwannoma cell death in both RT4 cell line and human primary cells. Importantly, we show, for the first time, that ART-induced cell death is largely dependent on necroptosis. Our data suggest that ART has great potential in schwannoma chemotherapy, especially when used in synergy with an apoptosis-inducing drug and/or an autophagy-inhibitory drug.

## Results

### The effect of ART on schwannoma cell death

**Figure 1:**
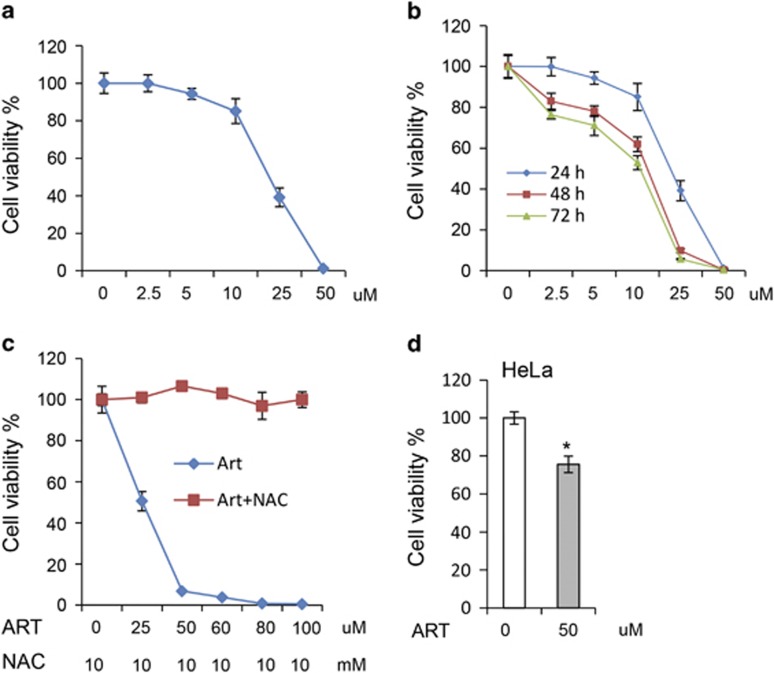
ART sufficiently induces schwannoma cell death that is reversed by ROS scavenger. (**a**) RT4 schwannoma cells were treated with ART for 20 h at indicated concentrations. Cell viability was measured with ATP-based Celltiter Glo kit (Promega). Data are shown as mean±S.D. (**b**) RT4 schwannoma cells were treated with ART at indicated concentrations for 24, 48 and 72 h, respectively. Cell viability was measured as above. Data are shown as mean±S.D. (**c**) RT4 schwannoma cells were treated with ART at indicated concentrations, in the presence or absence of 10 mM NAC for 20 h. Cell viability was measured. Data are shown as mean±S.D. (**d**) HeLa cells were treated with ART at indicated concentrations for 20 h. Cell viability was measured. Data were shown as mean±S.D. *P<0.05

To investigate whether ART can effectively kill schwannoma cells, we first tested the effects of ART on RT4 schwannoma cell death induction with a series of concentrations of ART (Figure 1a). Our data show that ART effectively killed RT4 schwannoma cells at 25 *μ*M, and the cells were almost 100% killed at the concentration of 50  *μ*M in 24 h (Figure 1a).

We further established the time-course effect of ART on cell death (Figure 1b). Taken together, these results demonstrate that ART is capable of killing RT4 schwannoma cells. We confirmed that the cell death was dependent on ROS since ROS scavenger, n-acety-cysteine (NAC), fully rescued the cell death effect of ART (Figure 1c). In contrast, ART exerted milder toxicity to HeLa cells in these conditions (Figure 1d). Table 1 shows that ART has cell killing effects on different cell types, however, RT4 schwannoma cells appeared to be more sensitive to ART among the cells.

**Figure 2:**
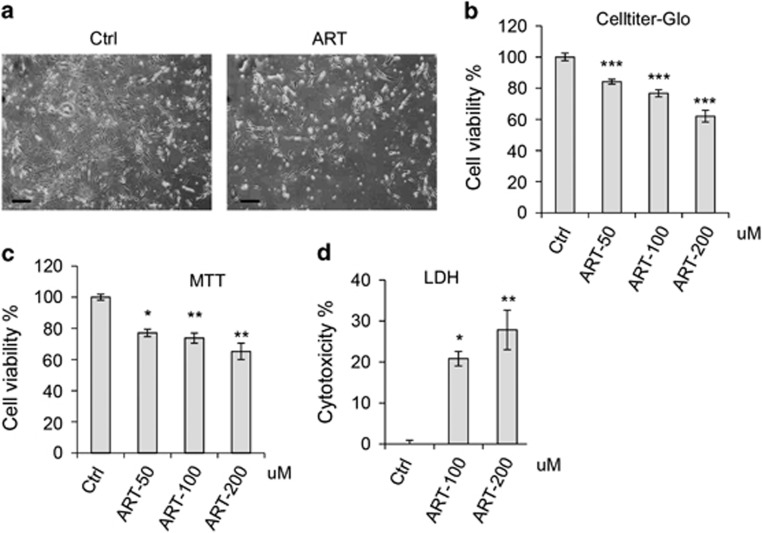
ART induces cell death in human primary schwannoma cells. (**a**) Human primary schwannoma cells were treated with ART (200 *μ*M). Phase-contrast cell images were acquired with a microscope. Scale bar: 200 *μ*m. To quantify cell viability/toxicity, human primary schwannoma cells were incubated with DMSO (control) or ART for 24 h. (**b**) Cell viability was measured using the Cell Titer-Glo Luminescent cell viability Assay. The cell viability in DMSO control was set as 100, and the relative values were computed in ART-treated samples. (**c**) Cell viability was measured using MTT assay. The viability in DMSO control was set as 100, and the relative values were computed in ART-treated samples. (**d**) Cytotoxicity was measured using the CytoTox-Fluor Cytotoxicity Assay. Cytotoxicity in DMSO control was set as 0. Data are shown as mean±S.E.M. *P<0.05; **P<0.01; ***P<0.001

ART appeared to be efficacious in killing RT4 schwannoma cells. We thus further tested whether the drug was effective in treating human primary schwannoma cells. Interestingly, ART was shown to kill human primary schwannoma cells effectively (~37%) (Figure 2a), although higher dose of the drug was needed for the killing compared with RT4 cells (Figure 2b). The effect of ART on cell death induction was tested in multiple assays, including viability assays and the cytotoxicity assay (Figures 2b–d).

### Apoptosis does not determine ART-induced cell death

**Figure 3:**
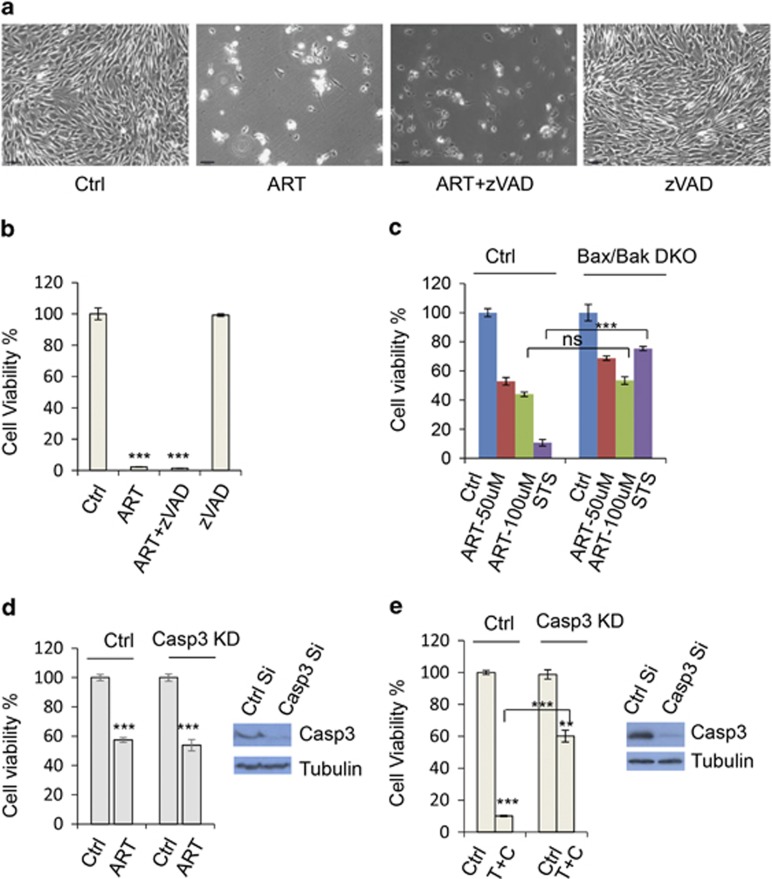
Apoptosis does not determine ART-induced cell death. (**a**) RT4 schwannoma cells were treated with DMSO, ART (50 *μ*M), ART (50 *μ*M)+zVAD (20 *μ*M) or zVAD (20 *μ*M). Images were acquired with contrast microscopy. Scale bar: 100 *μ*m. (**b**) Cell viability was measured. Data are shown as mean±S.D. (**c**) WT or Bax/Bak DKO MEFs were treated with ART at indicated concentrations, respectively. Meanwhile, apoptosis inducer staurosoporine (STS, 1 *μ*M) was used to treat WT or Bax/Bak DKO MEFs. After 20 h, cell viability was measured. Data are shown as mean±S.D. ***P<0.001; ns: not significant. (**d**) Control siRNA or caspase 3 siRNA was transfected into RT4 cells. Cells were treated with ART (25 *μ*M). Cell viability was measured with Cell Titer-Glo Luminescent cell viability assay. Data are shown as mean±S.D. ***P<0.001. Western blot was used to test caspase-3 knockdown effectiveness. (**e**) RT4 schwannoma cells were knocked down with control siRNA or caspase-3 siRNA. After 40 h, cells were treated with control (DMSO) or TNF (30 ng/ml)+cycloheximide (CHX, 30 *μ*M) as indicated for further 16 h. Cell viability was measured with Cell Titer-Glo luminescent assay. Data are shown as mean±S.D. **P<0.01; ***P<0.001

It is established that apoptosis is caspase-dependent cell death.^14^ We sought to know whether ART-induced cell death was caspase dependent in RT4 cells or whether apoptosis was a determinant for ART-induced cell death. To this end, the cells were treated with ART in the presence or absence of pan caspase inhibitor z-VAD-fmk (zVAD).^31^ Surprisingly, cell death induced by ART was not influenced by zVAD (Figures 3a and b). This suggests that ART-induced cell death is not determined by apoptosis.

Apoptotic pathways are well conserved across metazoans. This nature allows us to explore apoptosis in ART-induced cell death across different cell types from various species with exploitation of their unique genetic makeups. To validate that the cell death is independent of apoptosis, we treated Bax/Bak double knockout (DKO) mouse embryonic fibroblasts (MEFs) that do not undergo mitochondria-dependent apoptosis, which we used in our earlier study,^32^ and tested whether Bax/Bak DKO MEFs underwent cell death induced by ART. Figure 3c shows that the apoptosis-inducing drug staurosporine (STS)^33^ sufficiently killed wild-type (WT) MEFs but not Bax/Bak DKO MEFs. However, ART killed Bax/Bak DKO MEFs largely as effectively as WT MEFs (Figure 3c), further indicating that apoptosis is not a main factor contributing to ART-induced cell death.

ART has been suggested to induce apoptosis in tumour cells by a number of studies (reviewed in Lai *et al.*^34^).^35, 36, 37^ We thus further confirmed whether ART induced apoptosis in our system. It is well known that caspase 3 (casp-3) as an executioner is essential in apoptosis.^14^ Interestingly, the apoptosis hallmark casp-3 cleavage did not occur in RT4 cells and COLO-205 colon adenocarcinoma cells treated with ART, while casp-3 is activated in ART-treated HeLa cells (Supplementary Figure S1). We also confirmed that ART induced PARP cleavage, a marker of apoptosis (Supplementary Figure S2a).^38,39^ Consistently, immunostaining showed that casp-3 was activated by ART treatment in HeLa cells (Supplementary Figure S2b). These data suggest that depending on cell type, apoptosis may or may not occur in cells treated with ART. Figure 3d shows that casp-3 knockdown did not affect ART-induced cell death in RT4 cells, further indicating that apoptosis is not required for ART-induced RT4 schwannoma cell death. Similarly, casp-3 knockdown did not significantly reduce the cell death induced by ART in HeLa cells (Supplementary Figure S3). By contrast, caspase-3 knockdown ameliorated the toxicity caused by TNF+cycloheximide (CHX) treatment in the cells (Figure 3e), which has been established to induce apoptosis in various cells.^40,41^ Collectively, these data indicate that apoptosis does not occur in ART-treated RT4 schwannoma cells. Although apoptosis appears to occur in HeLa cells when treated with the drug, it may not be a major mechanism underpinning ART-induced cell death. In these cells, the cell death may be merely associated with apoptosis, but not determined by apoptosis.

### ART induces necroptosis in schwannoma cells

**Figure 4:**
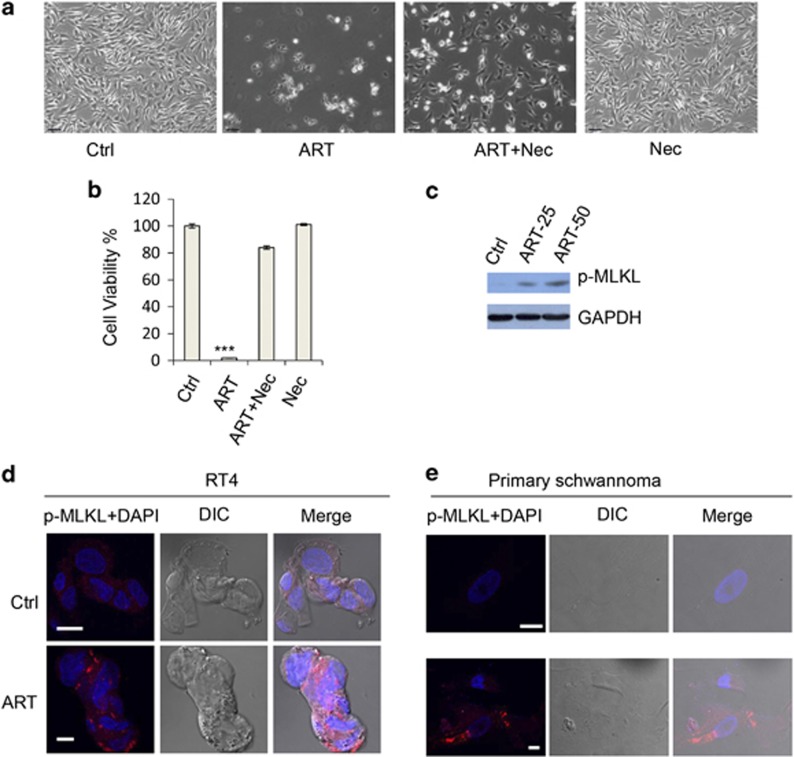
ART induces necroptosis in schwannoma cells. (**a**) RT4 schwannoma cells were treated with vehicle, ART (50 *μ*M), ART (50 *μ*M)+Nec (20 *μ*M) or Nec (20 *μ*M). Images were acquired with contrast microscopy. Scale bar: 100 *μ*m. (**b**) Cell viability was measured. Data are shown as mean±S.D. ***P<0.001. (**c**) RT4 cells were treated with DMSO and ART (25 or 50 *μ*M) for 20 h. Cells were lysed and subjected to SDS-PAGE and immunoblot with indicated antibodies. (**d**) RT4 cells were treated with DMSO and ART (25 *μ*M) for 20 h. Cells were then fixed and stained with p-MLKL. The images were acquired with confocal microscopy. (**e**) Human primary schwannoma cells were treated with DMSO and ART (100 *μ*M) for 20 h. Cells were then fixed and stained with p-MLKL. The images were acquired with confocal microscopy

Programmed necrosis or necroptosis is an alternative cell death in addition to apoptosis. As necroptosis is frequently associated with mitochondrial ROS generation,^42,43^ we assessed whether ART-induced cell death was necroptosis related by testing whether necrostatin 1 (Nec), a selective inhibitor of RIP1 that is essential for RIP1-RIP3-dependent necroptosis,^16,44,45^ could inhibit the cell death triggered by ART. Remarkably, the RT4 cell death was largely suppressed by Nec (Figures 4a and b), suggesting that necroptotic pathways have pivotal roles in ART-induced cell death. However, Nec did not exhibit any protective effect on the cell death induced by STS (Supplementary Figure S4a), which has been established to mainly induce apoptosis.^46^ These data further suggest that Nec specifically inhibits ART-induced necroptosis. Recently, Wang's group reported that phosphorylation of MLKL at threonine 357 (T357) and serine 358 (S358) is a hallmark of necroptosis,^47,48^ thus necroptosis can be detected by the phospho-MLKL antibody (p-MLKL). We tested whether necroptosis occurred when cells were induced by ART, and immunoblot confirmed that ART treatment induced MLKL phosphorylation (Figure 4c). Consistently, we observed that ART induced the necroptotic morphology decorated by p-MLKL in RT4 schwannoma cells (Figure 4d) and primary schwannoma cells (Figure 4e). Likewise, necroptosis was also observed in COLO-205 and HeLa cells (Supplementary Figure S4b and c). Collectively, these data suggest that necroptosis is induced by ART, which is crucial in mediating ART-induced cell death.

**Figure 5:**
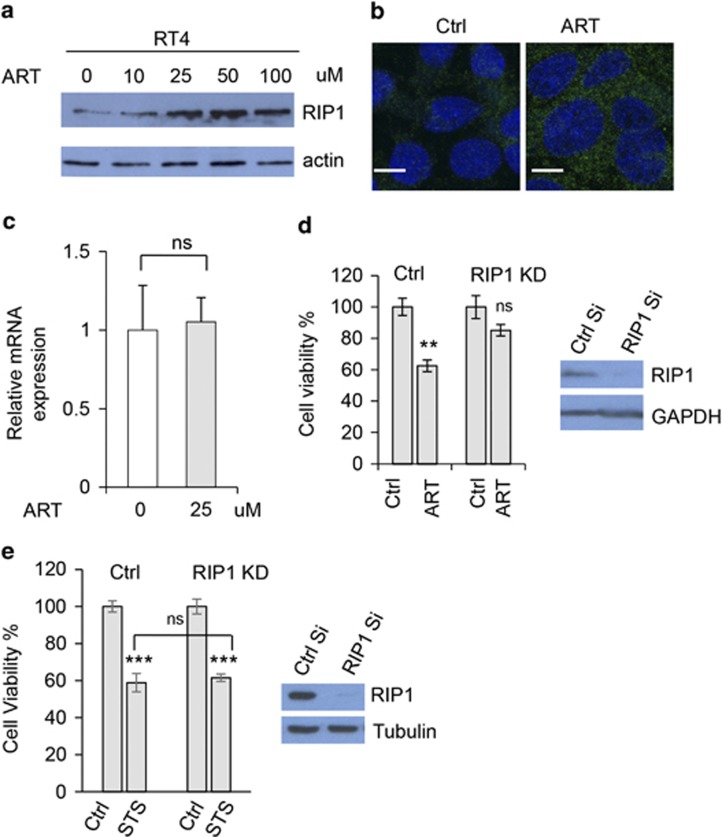
RIP1 may mediate ART-induced necroptosis in schwannoma cells. (**a**) RT4 cells were treated with ART at indicated concentrations. After 20 h, cells were harvested and cell lysates were subjected to SDS-PAGE, and blots were probed with anti-RIP1 and actin antibodies. (**b**) RT4 cells were treated with ART (25 *μ*M). After 24 h, cells were fixed and stained with anti-RIP1 antibody. Scale bar: 20 *μ*m. (**c**) RT4 cells were treated with control or ART (25 *μ*M) for 20 h. RNA was isolated from the cells, and subjected to analysis by qRT-PCR to detect the expression of RIP1 mRNA. The mean±S.D. of relative levels (normalized to GAPDH) from three independent experiments is shown. ns: not significant. (**d**) Control siRNA or RIP1 siRNA was transfected into RT4 cells. After 48 h, cells were treated with ART (25 *μ*M) for 20 h, and cell viability was measured with Cell Titer-Glo Luminescent cell viability assay. Data are shown as mean±S.D. **P<0.01. Western blot was used to test RIP1 knockdown effectiveness. (**e**) RT4 schwannoma cells were knocked down with control siRNA or RIP1 siRNA. After 40 h, cells were treated with control (DMSO) or staurosporine (STS, 0.5 *μ*M) for 15 h. Cell viability was measured with MTT assays. Data are shown as mean±S.D. ns: not significant; ***P<0.001

RIP1-RIP3 signalling has been shown to be essential in regulating necroptosis,^15, 16, 17^ thus we tested whether the protein levels in the pathway altered in ART-treated RT4 schwannoma cells. Interestingly, we found that RIP1 levels enhanced in the cells treated with the increasing concentrations of ART (Figure 5a). Consistently, immunochemistry also showed that RIP1 levels increased in the cells treated with ART (Figure 5b). However, qPCR shows that RIP1 mRNA levels were not regulated by ART (Figure 5c), suggesting that ART may upregulate RIP1 in protein level. By contrast, apoptosis inducer STS did not increase RIP1 levels as ART did (Supplementary Figure S5a), and ART did not appear to overtly alter the levels of apoptosis-related proteins (Supplementary Figure S5b). To test whether RIP1 levels are important in ART-induced cell death, we knocked down RIP1 and observed that RIP1 knockdown largely reduced the sensitization of the cells to ART treatment (Figure 5d), in contrast to the effect of casp-3 knockdown in the cell death (Figure 3d). However, RIP1 knockdown did not have any effects on STS-induced apoptosis (Figure 5e). These data further show that necroptosis is effectively required for the cell death and RIP1 is critical in mediating ART-induced necroptosis.

### Autophagy is inhibited by ART

**Figure 6:**
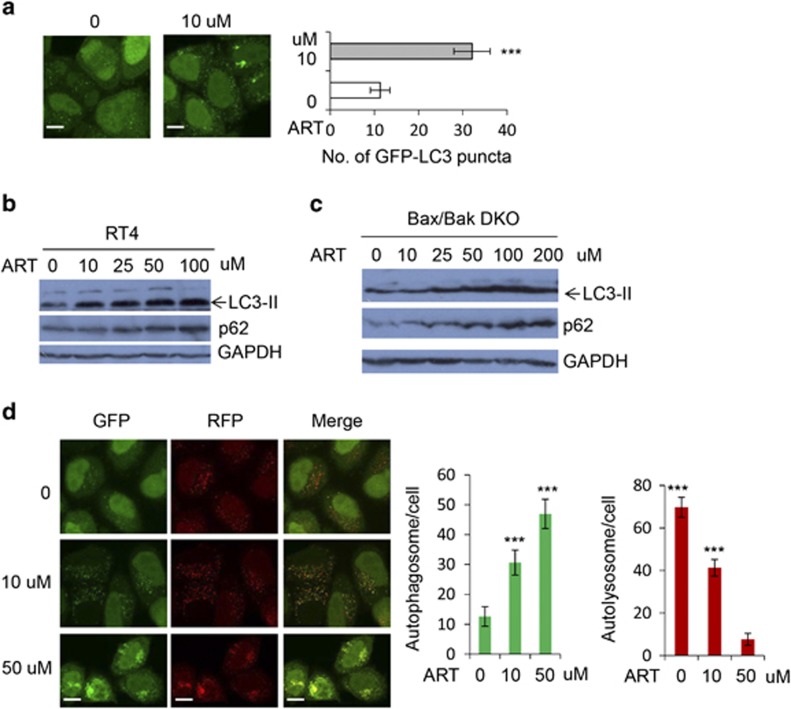
ART impairs autophagy. (**a**) GFP-LC3 stably expressing HeLa cells was treated with ART. GFP-LC3 vesicles were scored with Cellomics microscopy. Data are shown as mean±S.D. Scale bar: 20 *μ*m. (**b** and **c**) RT4 cells (**b**) or Bax/Bak DKO MEFs (**c**) were treated with ART at indicated concentrations. After 20 h, cells were harvested and cell lysates were subjected to SDS-PAGE, and blots were probed with anti-p62, LC3 or GAPDH antibodies. (**d**) GFP-mRFP-LC3-stably expressing HeLa cells were treated with ART for 24 h. Cells were fixed. Images were acquired with a confocal microscope. GFP and RFP vesicles were scored with Cellomics microscopy. Autophagosome number (green) and autolysosome number (red minus green) were evaluated. The numbers of autophagosomes (green vesicles) and autolysosomes (red vesicles minus green vesicles) were assessed with Cellomics microscopy. Data are shown as mean±S.D. ***P<0.001. Scale bar: 20 *μ*m

Auotphagy is a bulk lysosomal degradation system that mediates the clearance of long-lived toxic proteins and damaged organelles.^49^ In addition to apoptosis and necroptosis, cell death may also be regulated by autophagy since autophagy is now believed to have cytoprotective roles in cells.^50^ ART generates ROS in cells, which in turn can regulate autophagy.^51^ Therefore, we asked whether ART modulates autophagy. It is well known that autophagosome numbers correlate with the numbers of LC3-positive vesicles or the levels of the autophagosome-associated protein LC3-II.^52^ We observed that ART significantly increased autophagosome accumulation by measuring GFP-LC3 vesicles (Figure 6a) and LC3-II levels in RT4 and Bax/Bak DKO MEFs (Figures 6b and c).

To test whether autophagosome accumulation by ART is attributed to increased autophagosome synthesis or impaired autophagosome-lysosome fusion (thereby inhibiting autophagosome clearance), we employed GFP-mRFP-LC3 stably expressing cells for GFP-mRFP-LC3 vesicle analysis. This allows us to monitor autophagosome synthesis and autophagosome-lysosome fusion by labelling autophagosomes (green and red) and autolysosomes (red), since low lysosomal pH quenches GFP more quickly.^32,53^ We found that autophagosome number increased, while autolysosome number significantly decreased in the cells treated with ART (Figure 6d). These data suggest that ART inhibits autophagy by impairing lysosomal or autolysosomal function, leading to autophagosome accumulation. Consistently, we observed that ART inhibits autophagic flux/activity since the autophagic substrate p62 accumulated when the cells were treated with ART (Figures 6b and c). Collectively, these data suggest that ART-mediated autophagy inhibition and autophagosome accumulation may contribute to ART-induced cell death since autophagy activity is important for cell survival.^54^

### Combination treatment of ART and chloroquine can enhance the death of human primary schwannoma cells

**Figure 7:**
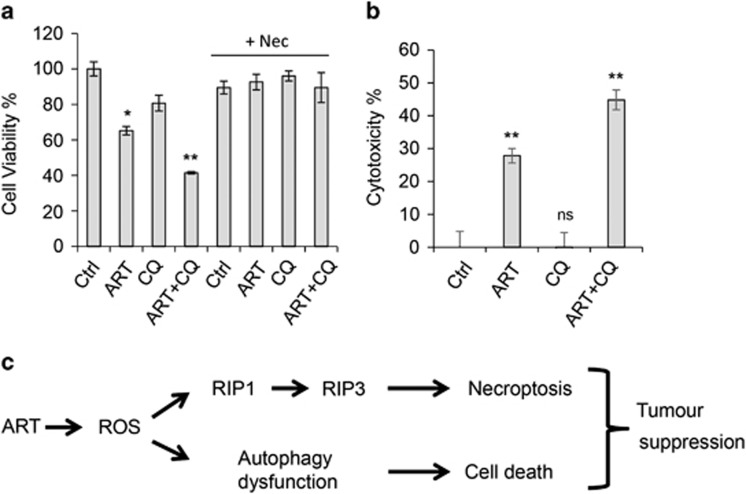
Chloroquine (CQ) enhances ART's effect in killing primary schwannoma cells. Human primary schwannoma cells were seeded at a density of 3 000 cells/well to 96-well plates and incubated with DMSO (control), CQ (25 *μ*M), ART (200 *μ*M) or a combination of CQ and ART for 24 h. The cells were also treated with Nec (20 *μ*M), Nec+CQ, Nec+ART or Nec+CQ+ART as indicated. Both cell viability and cytotoxicity were assessed. All graphs represent the mean±S.E.M. (**a**) Cell viability measured using MTT assay. The DMSO control is considered 100% viability. (**b**) Cytotoxicity measured using the CytoTox-Fluor Cytotoxicity Assay. The DMSO control is considered 0 cytotoxicity. *P<0.05; **P<0.01. (**c**) The double effects of ART in inducing cell death. ART induces cell death mainly via necroptotic pathways, and autophagy inhibition induced by ART may also contribute to the cell death. Apoptosis is associated with, but not determining the cell death

The data in Figure 6 suggest that ART-induced cell death may also attribute to autophagy inhibition in addition to necroptosis. We then directly tested whether autophagy inhibition alone induced primary schwannoma cell death. Chloroquine (CQ) is an approved anti-malarial drug and it also shows autophagy inhibition by blocking lysosomal activity.^55^ Interestingly, autophagy inhibition by CQ only induces modest cell death in the primary cells. We investigated whether combining treatments of ART and CQ could enhance the death of primary human schwannoma cells. Importantly, CQ increased the cell death induced by ART when added in combination (Figure 7a). Interestingly, Nec blocked ART or ART+CQ-induced cell death (Figure 7a). This confirms that necropotosis is required for ART-induced cell death. The results were consistent across multiple assays (Figures 7a and b). Like ART, CQ is also an anti-malarial drug that has been suggested to display some cytotoxic activity, although CQ alone only shows minimal toxicity in human primary schwannoma cells. Given that CQ is a well-documented medicine in clinic, the combinatory treatment of ART and CQ is a promising strategy to treat schwannomas.

Our observations suggest that ART is efficacious in inducing RT4 schwannoma cell death. Interestingly, ART largely induces necroptosis rather than apoptosis, and RIP1 levels are markedly enhanced by ART (Figure 5). ART also triggers autophagosome accumulation by inhibiting autophagic activity, likely contributing to the cell death. Figure 7c summarizes these observations and the proposed mechanisms underlying ART-induced cell death.

## Discussion

ART has been well documented in drug safety and efficacy in anti-malarial therapy, and it has been attractive as a cancer drug candidate due to its selective toxicity to cancer cells and low toxicity to normal cells. Previous studies have shown that ART has significant anti-tumour and anti-angiogenesis effects *in vivo* and *in vitro*.^10, 11, 12, 13^ There is a special need for new drug treatment in low grade tumours. A genetically well-defined group of low grade tumours are merlin-deficient tumours, and schwannomas have been used as a model for the group of tumours.^26,56,57^ It is unknown whether this drug is useful in schwannoma drug therapy, thus it is important to study the efficacy of ART in cell death induction of schwannomas as a model for merlin-deficient tumours and its role in cell death. Moreover, the mechanisms underlying ART-induced cell death are poorly understood, although it has been proposed that the drug induces cell death by apoptotic pathways.^34^

Our data demonstrate that ART is effective in cell death induction of RT4 schwannoma cells and human primary schwannoma cells. Previously, studies suggested that ART-induced cell death is apoptosis dependent, however, the conclusion was not tested with an apoptosis inhibitor or in cells where apoptosis is deficient.^35, 36, 37^ Unexpectedly, our current study shows that ART appears to solely induce RIP1-dependent necroptosis in schwannoma cells, although it induces both necroptosis and apoptosis in HeLa cells where apoptosis may be merely associated with ART-induced cell death rather than determinant for the cell death since casp-3 knockdown does not effectively reduce cell death induced by ART in the cells (Supplementary Figure S3). ART is competent in killing Bax/Bak DKO MEFs that are apoptosis defective (Figure 3). These data further suggest that apoptosis is not a major mechanism for the cell death after ART treatment. It is not clear how ART increases RIP1 levels. We postulate that ROS production from ART may compromise the ubiquitin-proteasome system that mediates RIP1 degradation, thus potentially enhancing RIP1 protein levels. We first demonstrate that ART-induced cell death is largely dependent on necroptosis rather than apoptosis in RT4 schwannomas and other cell types. This is important because it could lead us to understand that ART's efficacy can vary in different cell types, and offer us the knowledge on the application of the drug in the cells where necroptosis is competent. While using ART, other agents could be used to trigger apoptosis to maximize the efficacy of treatments.

In addition, we demonstrated that ART inhibits autophagy, and ART-led autophagy inhibition may also contribute to its induced cell death. Hamacher-Brady *et al.*^18^ showed that ART induces cell death *via* lysosomal ROS production in breast cancer cells, but it is enigmatic that lysosomotropic agent CQ or Bafilomycin A1 prevents ART-induced cell death in the cells. Interestingly, we found that autophagy inhibitor CQ significantly enhances ART efficacy in killing human primary schwannoma cells. These findings suggest that the combinatory treatment of CQ and ART needs to be further investigated for schwannoma drug therapy, given that both CQ and ART as mature malarial first-line medicines have proved safe clinically. Thus, this study highlights a new therapeutic implication on drug treatment for *NF2* as well as other tumours/cancer.

## Materials and Methods

### Antibodies and reagents

Rabbit polyclonal antibodies were anti-LC3 (1 : 10 000; Novus Biologicals, Cambridge, UK), anti-caspase 3 (1 : 1000; Cell Signaling, Hitchin, UK), phospho-T357-S358 MLKL (1 : 1000; Abcam, Cambridge, UK), anti-active caspase 3 (1 : 1000; Cell Signaling), anti-PARP (1 : 1000; Promega, Southampton, UK), RIP1 (1 : 1000; Cell Signaling), Bcl-xL (1 : 1000; BD, Oxford, UK), Bim (1 : 1000; Cell Signaling), Bax (1 : 1000; Cell Signaling), caspase 8 (1 : 1000; Cell Signaling) and anti-actin (1 : 2000; Sigma, Gillingham, UK). Anti-mouse monoclonal antibodies were anti-GAPDH (1 : 5000; Ambion, Warrington, UK), anti-p62 (1 : 1000; BD) and anti-tubulin (1 : 5000; Sigma). ART, CQ and Nec were purchased from Sigma. zVAD-fmk (zVAD) was a product of Merck (Feltham, UK). TNF*α* was purchased from Invitrogen (Paisley, UK). CHX and STS were from Sigma. All control siRNAs and siRNAs against caspase 3 and RIP1 were from Dharmacon (Lafayette, CO, USA).

### Cell culture

Bax/Bak DKO MEFs were kindly offered by Dr Christoph Borner (University of Freiburg, Germany). HeLa cells and Bax/Bak DKO MEFs were cultured with standard methods in DMEM supplemented with 10% FCS (Sigma). RT4 schwannoma cells were purchased from Sigma and cultured in DMEM with 10% FCS.

### siRNA transfection

Cells were split 1 day before transfection to 50% confluence and left overnight in antibiotic-free DMEM containing 10% FBS. siRNAs were transfected with Lipofectamine 2000 (Invitrogen) according to the manufacturer's instructions. The final concentration of siRNAs was 100 nM. Non-targeting siRNA was the control siRNA. Cells were maintained in 10% FBS DMEM containing no antibiotics for 48 hours after transfection.

### Schwannoma primary cell isolation and culture

Ethical approval is granted and patients gave consent with the usage of the tumour samples to isolate of human primary schwannoma cells. The methods were described by Rosenbaum *et al.*^58^ Briefly, schwannomas were surgically removed under local anaesthesia, and were then preincubated for 1–7 days in incubation medium (DMEM plus 10% FBS, 500 U/ml penicillin/streptomycin, 0.5 *μ*M Forskolin, 2.5 *μ*g/ml Amphotericin B) in 10% CO_2_ and then dissected into 1-mm-long pieces in DMEM with 10% FCS containing 500 U/ml penicillin/streptomycin, 160 U/ml collagenase type I (Sigma) and 1.25 U/ml dispase grade I (Roche, West Sussex, UK). Tissue pieces were incubated in proteolytic enzymes for 24 h before they were dissociated by trituration with a narrowed Pasteur pipette. Cell suspension was added to a 50-ml Falcon tube. Cells were collected and resuspended in proliferation medium: DMEM with 10% FCS, 500 U/ml Pen/Strep, 0.5 *μ*M forskolin (Tocris, Abingdon, UK), 2.5 *μ*g/ml Amphotericin B, 10 nM b1-heregulin (R&D System, Abingdon, UK) and 2.5 *μ*g/ml insulin (Sigma). Cells were seeded into 96-well plates (Greiner Bio-one, Stonehouse, UK), coated with 1 mg/ml poly-l-lysine (Sigma) and 4 *μ*g/ml natural mouse laminin (Life Technologies, Paisley, UK), at a density of 3000 cells/well. Proliferation medium was changed every 3–4 days and cells were passaged when confluent.

### Cell viability assay

#### ATP assays

Cell survival was determined with the Cell Titer-Glo Luminescent cell viability Assay kit (Promega) to measure ATP levels according to the manufacture's instruction. Briefly, 100 *μ*l of Cell Titer-Glo reagent was added to the culture medium. Cells were placed on a shaker for 5 min and then incubated at room temperature for 10 min. The SPECTRA Max M5 reader (Molecular Devices, Workingham, UK) was used for Luminescent reading.

#### MTT assays

For the MTT viability assay, 10 *μ*l of a 12 mM MTT stock solution (Invitrogen) was added to the culture medium and incubated at 37°C for 4 h. Medium was replaced with 100 *μ*l DMSO and placed on a plate shaker for 10 min. Absorbance was read at 562 nm and a reference measurement at 650 nm. Readings were performed with the TECAN GENios V4.62-07/01 microplate reader (Tecan, Reading, UK) with XFLUOR4 Version V 4.51 software (Tecan).

### Cytotoxicity assay

Cytotoxicity was measured using the Promega CytoTox-Fluor Cytotoxicity Assay kit as per the manufacturers' instructions. Briefly, 10 *μ*l of reagent was added to the culture medium and then incubated at 37°C for 3 h. Plates were put on a shaker for 5 min before fluorescence was measured at 485nm_ex_/535nm_em_ with the TECAN GENios V4.62-07/01 microplate reader with XFLUOR4 Version V 4.51 software.

### GFP-mRFP-LC3 assay

HeLa cells stably expressing GFP-RFP-LC3 were treated with ART at the indicated concentrations. After 24 h, cells were fixed in 2% PFA for 5 min. Cellomics (Arrayscan VTI) was used to score green and red vesicles. Green vesicles are considered to be autophagosomes and red vesicles are considered to be both autophagosomes and autolysosomes. The number of autolysosomes was achieved by subtracting the number of green vesicles from that of the red vesicles.

### Analysis of autophagosomes/vesicles

In experiments requiring a precise assessment of vesicle number, the number of vesicles per cell in GFP-positive cells was determined. Approximately 100 cells per sample were counted for triplicate samples, as described previously.^32^ All coverslips were scored with the observer blinded to the identity of the slides.

### Immunocytochemistry

After transfection, cells were fixed with 4% paraformadehyde for 10 min after washing with phosphate-buffered saline (PBS) twice. The fixed cells were washed three times in PBS, then permeablized with 0.5% Triton in PBS for 10 min. Cells were blocked in blocking buffer (1% BSA, 1% heat inactivated goat serum in PBS) for 30 min at room temperature. Primary antibodies were incubated with cells overnight at 4°C. The secondary antibody was incubated for 30 min after washing three times (10 min, each). Cells were washed three times (10 min, each) after incubation with secondary antibodies, then mounted with DAPI (3 *μ*g/ml). Images were acquired on a Zeiss LSM710 META microscope (63x1.4NA planapochnomat oil immersion) (Carl Zeiss, Welwyn Garden City, UK).

### qRT-PCR analysis

RT4 cells were treated as indicated and RNA was isolated using TRIzol reagent following the manufacturer's instructions (Invitrogen). For qPCR analysis, 1ug RNA was reverse transcribed (Applied Biosystems, Paisley, UK) using the procedure of 25°C (10 min), 37°C (120 min) and 85°C (5 min). The resulting cDNA templates were subjected to qPCR using LightCycler 480 DNA SYBR Green I Master kit (Roche) with LightCycler 480 II system (Roche). GAPDH was used as a control to normalize the data. RIP1 primers (0.5 *μ*M): 5′-AATAGTTCTCGTGTTCAGATTGGA-3′ (forward) and 5′-AGTGTTGGTTGGTGGTTGT-3′ (reverse) (Sigma); GAPDH primers (0.5 *μ*M): 5′-ATCACTGCCACCCAGAAGAC-3′ (forward) and 5′-CAGTGAGCTTCCCGTTCAG-3′ (reverse) (Microsynth, Balgach, Switzerland).

### Statistics

*T*-test was used and *P*-values were determined by unconditional logistical regression analysis by using the general loglinear option of SPSS 9.1 software (SPSS, Chicago, IL, USA) (***P<0.001; **P<0.01; *P<0.05; NS, not significant).

## Figures and Tables

**Table 1:** The effect of ART on various cell types

**Cell Type**	**% Viability with ART 50 *μ*M**
RT4	0
HEK293T	45.3
Colo-205	51.1
SH5Y	71.3
HeLa	78

RT4, HEK293T, COLO-205, SH5Y and HeLa cells were treated with control (DMSO) or 50 *μ*M ART. After 20 h, cell viability was measured with MTT assays. The cell viability in 50 *μ*M ART was calculated in relation to control, in which the viability was set as 100%

## Abbreviations

ART: Artesunate
casp-3: caspase 3
CQ: chloroquine
MEFs: mouse embryonic fibroblasts
NF2: Neurofibromatosis type II
PCD: programmed cell death
ROS: reactive oxygen species
STS: staurosporine
zVAD: zVAD-fmk
