# Supplementary material for: Artesunate induces necrotic cell death in schwannoma cells
Source: Cell Death Dis. 2014 Oct 16;5(10):e1466–. doi: 10.1038/cddis.2014.434 (PMC4649524; doi:10.1038/cddis.2014.434)
Supplement: Supplementary Figures [file cddis2014434x1.pdf]

Fig S1

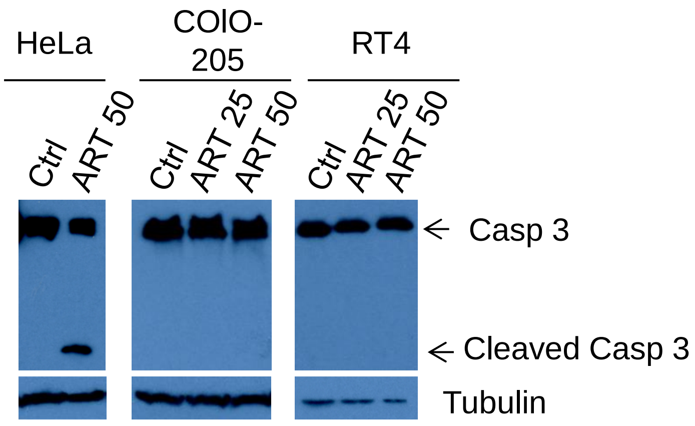
HeLa
COlO-205
RT4
Ctrl
ART 50
Ctrl
ART 25
ART 50
Ctrl
ART 25
ART 50
Casp 3
Cleaved Casp 3
Tubulin

Fig S2

a

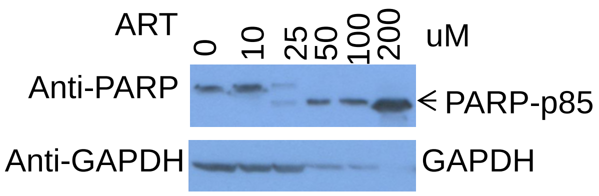
ART
0
10
25
50
100
200
uM
Anti-PARP
PARP-p85
Anti-GAPDH
GAPDH

b

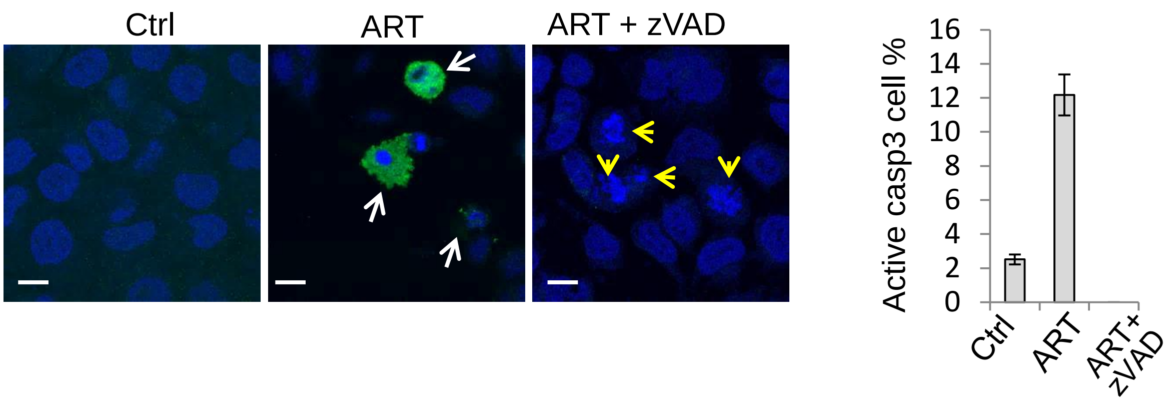
Ctrl
ART
ART + zVAD
Active casp3 cell %
16
14
12
10
8
6
4
2
0
Ctrl
ART
ART+ zVAD

Fig S3

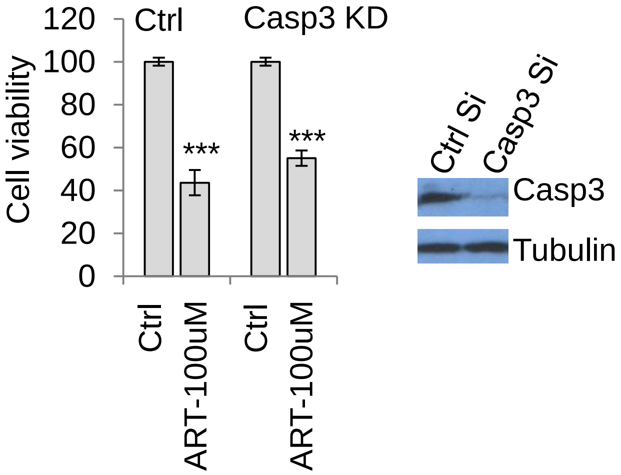
Cell viability
120
100
80
60
40
20
0
Ctrl
Casp3 KD
***
***
Ctrl
ART-100uM
Ctrl
ART-100uM
Ctrl Si
Casp3 Si
Casp3
Tubulin

Fig S4

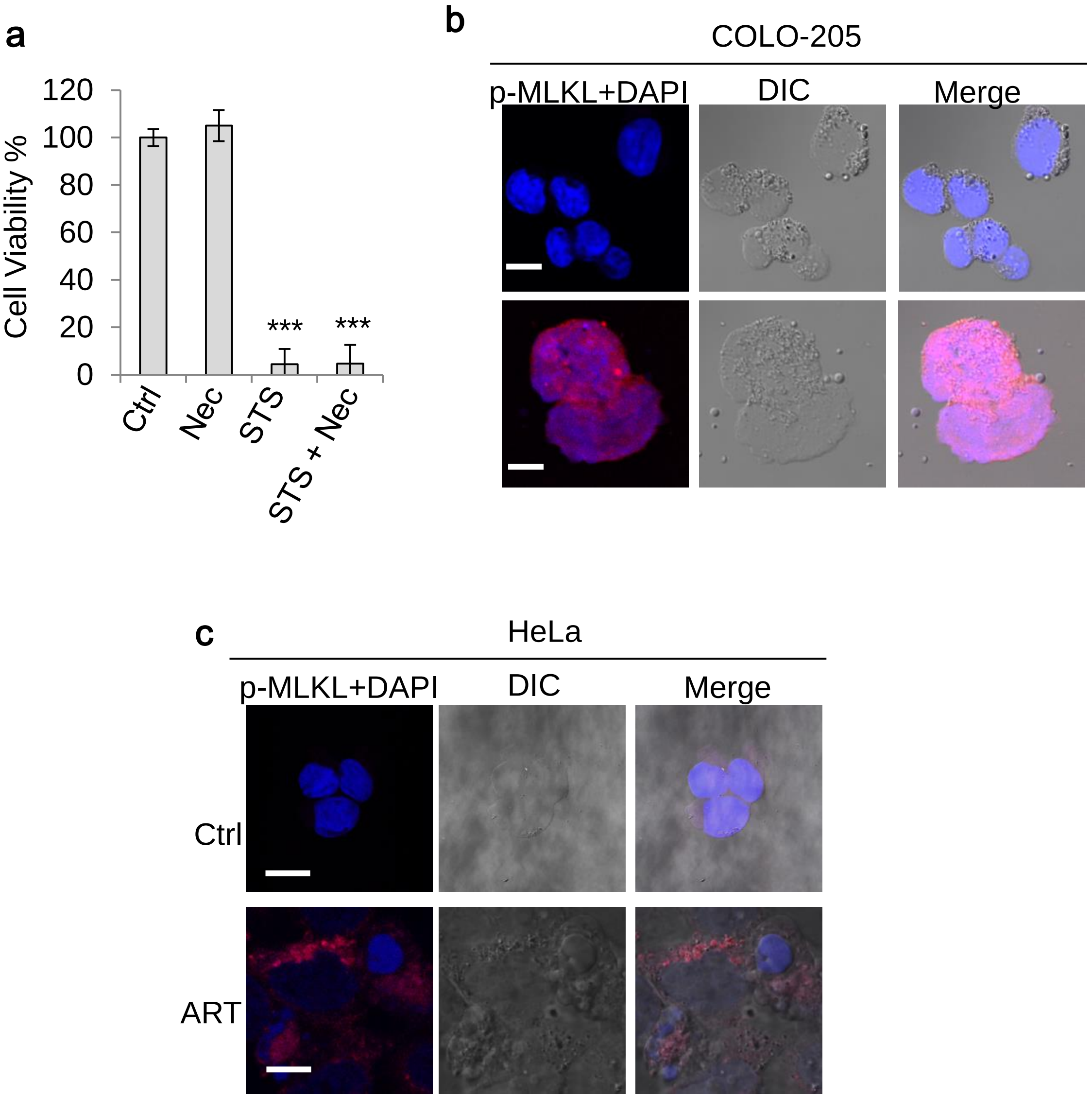
a
Cell Viability %
120
100
80
60
40
20
0
***
***
Ctrl
Nec
STS
STS + Nec
b
COLO-205
p-MLKL+DAPI
DIC
Merge
c
HeLa
p-MLKL+DAPI
DIC
Merge
Ctrl
ART

Fig S5

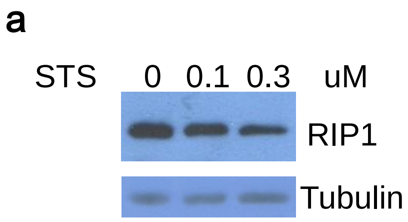
a
STS 0 0.1 0.3 uM
RIP1
Tubulin

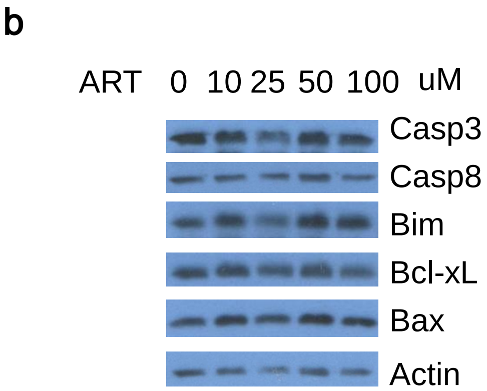
b
ART 0 10 25 50 100 uM
Casp3
Casp8
Bim
Bcl-xL
Bax
Actin

Supplementary figure legends

Fig S1. HeLa, COLO-205 or RT4 cells were treated with ART at the indicated concentrations (uM). Cells were then lysed and cell lysates were subjected to SDS-PAGE and blotted with anti-casp-3 antibody and anti-tubulin (loading control).

Fig S2. a. HeLa cells were treated with ART at the indicated concentrations. Cells were then lysed and cell lysates were subjected to SDS-PAGE and blotted with anti-PARP antibody and anti-GAPDH (loading control). **b.** HeLa cells were treated with ART (100 uM) for 24 hours. Cells were then fixed with 4% PFA and stained with active casp-3. The images were acquired by a confocal microscope. White arrows indicate active casp 3-positive cells; yellow arrows show abnormal nuclei in ART+zVAD-treated cells. Scale bar: 20 um. Active casp 3-positive cells were scored and the percentage was computed.

Fig S3. Control siRNA or caspase 3 siRNA was transfected into HeLa cells. Cells were treated with ART (100 uM). Cell viability was measured with Cell Titer-Glo Luminescent cell viability assay. Data are shown as mean±sd. ***: $P<0.001$. Western blot was used to test caspase-3 knockdown effectiveness.

Fig S4. a. RT4 cells were treated with control, Nec (20 uM), staurosporine (STS, 1 uM) and Nec (20 uM)+STS (1 uM) for 18 hours. Cell viability was measured with MTT assay. Data are shown as mean±sd. ***: $P<0.001$. **b.** COLO-205 cells were treated with DMSO or ART (50 uM) for 20 hours. Cells were then fixed and stained with p-MLKL. The images were acquired with a confocal microscope. **c.** HeLa cells were treated with DMSO and ART (50uM) for 20 hours. Cells were then fixed and stained with p-MLKL. The images were acquired with a confocal microscope.

Fig S5. a. RT4 cells were treated with STS at indicated concentrations. After 20 hours, cells were harvested and cell lysates were subjected to SDS-PAGE, and blots were probed with RIP1 and tubulin antibodies successively. **b.** RT4 cells were treated with ART at indicated concentrations. After 20 hours, cells were harvested and cell lysates were subjected to SDS-PAGE, and blots were probed with the antibodies indicated and actin antibody successively.
